# Estimates of the likely prophylactic effect of tamoxifen in women with high risk BRCA1 and BRCA2 mutations

**DOI:** 10.1038/sj.bjc.6600064

**Published:** 2002-01-21

**Authors:** S W Duffy, R M Nixon

**Affiliations:** Department of Mathematics, Statistics and Epidemiology, Imperial Cancer Research Fund, 61 Lincoln's Inn Fields, London WC2A 3PX, UK; MRC Biostatistics Unit, Institute of Public Health, Robinson Way, Cambridge CB2 2SR, UK

**Keywords:** breast cancer, chemoprevention, tamoxifen, BRCA1 mutation, BRCA2 mutation

## Abstract

The development of breast cancer control strategies in women at high genetic risk of breast cancer is an important issue. The likely benefit of chemopreventive approaches is of particular interest. Tamoxifen tends to be more effective in both prevention and treatment of oestrogen receptor positive tumours than oestrogen receptor negative. In this study, we combine the oestrogen-receptor specific effects of tamoxifen from randomized preventive or therapeutic trials with the oestrogen receptor status of tumours in BRCA1 and BRCA2 mutation positive women from published tumour surveys to obtain estimates of the likely effect of tamoxifen administration in mutation carriers. We used a simple two-stage procedure to estimate the benefit as a weighted average of the effect on oestrogen receptor positive tumours and oestrogen receptor negative, and using a more complex hierarchical modelling approach. Using the simple procedure and deriving the estimates of benefit from both primary prevention and therapeutic trials, we obtain an estimated reduction in risk of breast cancer from administration of tamoxifen in BRCA1 mutation positive women of 13% (RR=0.87, 95% CI 0.68–1.11). The corresponding estimated reduction in BRCA2 mutation positive women was 27% (RR=0.73, 95% CI 0.59–0.90). Using the more complex models gave essentially the same results. Using only the primary prevention trials gave smaller estimates of benefit in BRCA1 carriers but larger estimates in BRCA2, in both cases with wider confidence intervals. The benefit of prophylactic use of tamoxifen in BRCA1 mutation carriers is likely to be modest, and the effect in BRCA2 mutation carriers somewhat greater.

*British Journal of Cancer* (2002) **86**, 218–221. DOI: 10.1038/sj/bjc/6600064 www.bjcancer.com

© 2002 The Cancer Research Campaign

## 

In randomized trials, tamoxifen has been shown to be effective in treatment of oestrogen receptor positive (ER+) tumours but not of oestrogen receptor negative (ER−), in terms of prevention of recurrences, new primary tumours and fatality (Early Breast Cancer Trialists' Collaborative Group, 1998). One primary prevention trial in the USA found that tamoxifen substantially reduced incidence of ER+ tumours but had no such effect on ER− (Fisher *et al*, 1998). In sporadic breast cancer, the majority of tumours are ER positive (Early Breast Cancer Trialists' Collaborative Group, 1998), whereas the opposite is the case in cancers diagnosed in women with high risk mutations in the BRCA1 and BRCA2 genes (Johannsson *et al*, 1997; Armes *et al*, 1999).

In this study we synthesize the results on oestrogen receptor (ER) status from tumour series in mutation carriers with subgroup analyses by ER status in primary and secondary prevention trials of long-term tamoxifen use. From this, we derive an estimate of the likely preventive benefit of long-term tamoxifen administration in women with high risk BRCA1 and BRCA2 mutations.

## MATERIALS AND METHODS

First, three computerized literature searches augmented with studies brought to our attention by personal communication were performed: (1) To find surveys of ER status in breast cancer patients with a high risk mutation in the BRCA1 or BRCA2 gene. Seventeen such studies were found (Johannsson *et al*, 1997; Karp *et al*, 1997; Tirkkonen *et al*, 1997; Agnarsson *et al*, 1998; Eiriksdottir *et al*, 1998; Loman *et al*, 1998; Lynch *et al*, 1998; Osin *et al*, 1998; Robson *et al*, 1998; Verhoog *et al*, 1998; Wagner *et al*, 1998; Armes *et al*, 1999; Eisinger *et al*, 1999; Noguchi *et al*, 1999; Phillips *et al*, 1999; Tang *et al*, 1999; Chang *et al*, 2001). (2) To find randomized trials of tamoxifen administration for at least 3 years for primary prevention of breast cancer, with published results stratified by ER status. Two such trials were found (Fisher *et al*, 1998; Veronesi *et al*, 1998). One other prevention trial did not publish results by ER status (Powles *et al*, 1998). (3) To find randomized trials of tamoxifen administration for at least 3 years in breast cancer patients for prevention of recurrences or new primary breast cancers, with published results stratified by ER status. Five such studies were found (Delozier *et al*, 1986; Breast Cancer Trials Committee, 1987; Falkson *et al*, 1990; Tormey *et al*, 1992; Rutqvist *et al*, 1996).

Results of each of the above three types of study were first synthesized using random effects meta-analysis methods, details of which are given by Nixon and Duffy (2002). Results of the trials were then combined with those of the BRCA1 and BRCA2 tumour surveys in turn, as follows: let *R_−_* be the percentage reduction conferred by tamoxifen in ER− tumours, as observed in the combined randomized trials. Let *R*_+_ be the corresponding reduction in ER+ tumours. Let *p* represent the proportion of ER+ tumours estimated from the combined results of the BRCA1 tumour surveys. Then the percentage reduction in breast cancers to be expected from administration of tamoxifen to subjects with these mutations is


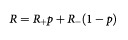


We estimated *R*_+_, *R_-_* and *R* twice, first using both the primary and secondary prevention trials, and second using the primary prevention trials only. We calculated a 95% confidence interval on R using the formula in Appendix 1. The procedures were then repeated using the proportion of ER+ tumours from the BRCA2 surveys.

Using the above two-stage estimation method, the 95% confidence interval depends on the assumption of independence of *R*_+_ and *R_−_,* which is not strictly true and may cause overestimation or underestimation of the standard error. We therefore also estimated *p*, *R*_+_, *R_−_* and *R* simultaneously in a hierarchical model with random effects estimation of trial results and tumour series results (Nixon and Duffy, 2002), using the Gibbs sampling algorithm for estimation, implemented in the Bayesian computer package BUGS (Gilks *et al*, 1994). This yields a 95% credible interval (the Bayesian analogue of a confidence interval) which takes into account the full uncertainty in all parameters simultaneously and does not assume independence of *R*_+_ and *R_−_*.

## RESULTS

Table 1

**Table 1:**
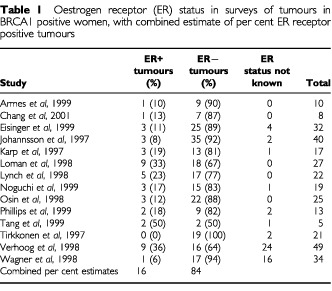
Oestrogen receptor (ER) status in surveys of tumours in BRCA1 positive women, with combined estimate of per cent ER receptor positive tumours

shows the individual and combined results of the tumour surveys of ER status in BRCA1 mutation carriers. The overall estimate of the proportion of ER+ tumours was 0.16 (95% CI 0.09–0.23). Table 2

**Table 2:**
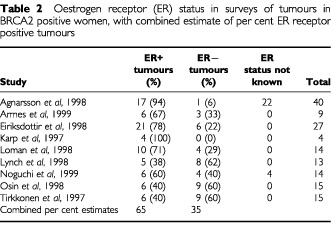
Oestrogen receptor (ER) status in surveys of tumours in BRCA2 positive women, with combined estimate of per cent ER receptor positive tumours

shows the corresponding results for BRCA2 mutation carriers, with an overall estimate of the proportion ER+ of 0.65 (95% CI 0.55–0.75).

Table 3

**Table 3:**
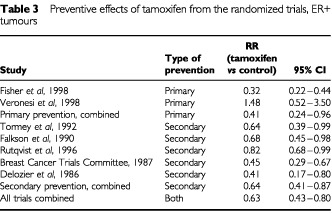
Preventive effects of tamoxifen from the randomized trials, ER+ tumours

shows the individual and combined effects of tamoxifen on incidence of ER+ cancers in the randomized trials. In the primary prevention trials, there was a significant overall reduction in incidence of 59% (RR=0.41, 95% CI 0.24–0.96). For the secondary prevention trials, the reduction was significant at 36% (RR=0.64, 95% CI 0.41–0.87). For all trials combined, the reduction was significant, at 37% (RR=0.63, 95% CI 0.43–0.80).

Table 4

**Table 4:**
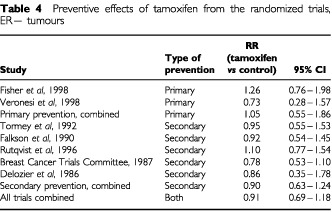
Preventive effects of tamoxifen from the randomized trials, ER− tumours

shows the results of the trials in terms of prevention of ER− tumours. In the primary prevention trials there was a non-significant excess of tumours of 5% (RR=1.05, 95% CI 0.55–1.86). In the secondary prevention studies, there was a non-significant reduction of 10% (RR=0.90, 95% CI 0.63–1.24). For all trials combined, there was a non-significant reduction of 9% (RR=0.91, 95% CI 0.69–1.18).

Table 5

**Table 5:**
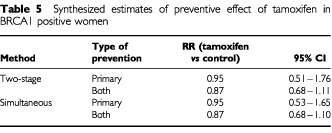
Synthesized estimates of preventive effect of tamoxifen in BRCA1 positive women

gives the results of combining the findings of the trials with those of the tumour surveys with BRCA1 mutation positive, by estimation method and type of studies used. There is no significant or sizeable non-significant reduction in incidence estimated from the primary prevention studies alone. When all trials are combined there is a modest estimated 13% reduction by both the two stage method (RR=0.87, 95% CI 0.68–1.11) and the simultaneous estimation method (RR=0.87, 95% CI 0.68–1.10), with *P*=0.2 in both cases.

The corresponding estimates for BRCA2 mutation positive women are given in Table 6

**Table 6:**
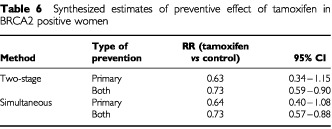
Synthesized estimates of preventive effect of tamoxifen in BRCA2 positive women

. The analyses using all trials both yield a significant 27% reduction. Analyses using only the primary prevention studies give a greater absolute reduction (36–37%), which just falls short of statistical significance (*P*=0.09) using the simultaneous estimation procedure (RR=0.64, 95% CI 0.40–1.08).

## DISCUSSION

The above suggests that any preventive benefit of tamoxifen in women positive for the high risk BRCA1 mutation is likely to be modest, but that a larger benefit of the order of a 25–35% reduction in incidence may be conferred in BRCA2 mutation carriers. This finding stems from the lesser effect of tamoxifen in prevention or treatment of ER− cancers, which are more common in BRCA1 mutation carriers.

The literature on this subject is inconclusive (see for example Eeles and Powles (2000)). There is some support for a substantial benefit of tamoxifen in prevention of contralateral tumours in mutation-positive breast cancer cases, from a retrospective case-control study (Narod *et al*, 2000). Simulation studies also suggest a secondary preventive effect (Schrag *et al*, 2000). Our results suggest a modest primary preventive effect, which is stronger in BRCA2 positive women than in BRCA1 positive. In principle, however, breast cancer risk in BRCA1 positive women should be amenable to hormonal manipulation, since it has been shown that oophorectomy reduces risk in such women (Rebbeck, 2000).

The modest effect, which we observed in BRCA1 mutation carriers, approaching statistical significance, and the clear benefit of the order of 25–35% in BRCA2, is for the most part consistent with the literature. The exception to this is the study of Narod *et al* (2000). This was a case–control study comparing tamoxifen history in bilateral breast cancer patients with a BRCA1 or BRCA2 mutation with that in unilateral patients, also with a mutation. The authors found a substantial protective effect of tamoxifen on contralateral disease in BRCA1 carriers, with a reduction in odds of 62%. This is admittedly the secondary preventive effect, but even so the difference in results is surprising. Our study has the advantage that the treatment effects are based on prospective randomized studies, but the disadvantage of having to make the assumption that the effect of tamoxifen is chiefly dependent on oestrogen receptor status. The advantage of the study of Narod *et al* (2000) is that it directly links breast cancer occurrence with tamoxifen history in women with BRCA1 and BRCA2 mutations. The disadvantage is its retrospective and non-randomized design. Since both studies have positive and negative aspects, it is difficult at this stage to see which is giving the correct answer.

The major assumption in this work is that the principal determinant of the benefit of tamoxifen is the oestrogen receptor status of the tumour being treated or prevented, and that mutation status only affects tamoxifen's performance via the ER status. The first of these seems to be true on the basis of the experimental evidence (Tables 3 and 4). The results of the trials are at least consistent with the second (Tables 3 and 4), but it cannot be verified or refuted for certain without a randomized trial of tamoxifen status in mutation positive women or a stratification of tumours diagnosed in existing prevention trials by mutation status. This would also resolve the question raised by the inconsistency of our results with those of Narod *et al* (2000). We understand that this stratification is under way in the NSABP-P1 study. In the Italian prevention trial (Veronesi *et al*, 1998), some work in preparation suggests that tamoxifen is effective in preventing hormone-dependent cancers, as judged by the presence of risk factors such as low parity and late age at first birth, and is not effective in prevention of non-hormone-dependent cancers, as judged by absence of reproductive risk factors but presence of a family history of breast cancer (P Boyle, personal communication).

In the above, we attempted to obtain the preventive effects by ER status directly from the primary research. For secondary prevention, we could have simply used the effects of 5 years' tamoxifen treatment from the Early Breast Cancer Trialists' Collaborative Group (1998). This gave a secondary preventive relative risk of 0.50 for ER+ tumours and 0.94 for ER−. Using these to give the combined estimated benefit from both primary and secondary prevention studies we would have obtained an estimated reduction of 13% (RR=0.87, 95% CI 0.70–1.08) in BRCA1 positive women and 35% (RR=0.65, 95% CI 0.55–0.76) in BRCA2 positive women. These are similar to the results in Tables 5 and 6.

The findings of this work have several implications for future work. Firstly, if studies are set up to determine the preventive effect of hormonal agents in women at high genetic or familial risk, relatively modest effects should be postulated in the study size calculations. Secondly, the preventive effect suggested by our work in BRCA1 positive women is not substantial and the subjects would still have a considerable residual absolute risk. For these women, it may be more profitable to explore non-hormonal prevention strategies. Finally, in recruitment of high risk women for hormonal chemoprevention trials, it might be prudent to use risk criteria based on hormonal rather than familial factors.

## APPENDIX 1

### Approximate confidence interval for the two-stage method

The square of the standard error of *R* is the variance of *R*, denoted *V(R)*.

where *C* represents the covariance. Noting that *V(p)* =*V(1-p)* and assuming *R*_+_, R_−_ and *p* are independent, the above can be estimated as

Using the delta method we can approximate the standard error of the logarithm of the relative risk *R* as

We then calculate the confidence interval on the log relative risk and retransform to the linear scale in the usual way.
